# Intracellular C5aR1 inhibits ferroptosis in glioblastoma through METTL3-dependent m6A methylation of GPX4

**DOI:** 10.1038/s41419-024-06963-5

**Published:** 2024-10-05

**Authors:** Xiangrui Meng, Zixuan Wang, Qingqing Yang, Yawei Liu, Yisu Gao, Hefei Chen, Ang Li, Rongqing Li, Jun Wang, Guan Sun

**Affiliations:** 1 Department of Neurosurgery, The Yancheng Clinical College of Xuzhou Medical University, The First People’s Hospital of Yancheng, Yancheng, China; 2 Yancheng Medical Research Center of Nanjing University Medical School, Yancheng First Hospital, Affiliated Hospital of Nanjing University Medical School, The First People’s Hospital of Yancheng, Yancheng, China; 3 Department of Radiation Oncology, The Affiliated Cancer Hospital of Nanjing Medical University, Jiangsu Cancer Hospital, Nanjing, China; 4 Postgraduate College, Xuzhou Medical University, Xuzhou, China; 5 Institute of Translational Medicine, Medical College, Yangzhou University, Yangzhou, China

**Keywords:** CNS cancer, Cell death, Epigenetics

## Abstract

Glioblastoma (GBM) is the most common primary intracranial malignant tumor. Recent literature suggests that induction of programmed death has become a mainstream cancer treatment strategy, with ferroptosis being the most widely studied mode. Complement C5a receptor 1 (C5aR1) is associated with both tumorigenesis and tumor-related immunity. However, knowledge regarding the role of C5aR1 in GBM progression is limited. In the present study, we observed significant upregulation of C5aR1 in glioma tissue. In addition, C5aR1 expression was found to be closely associated with patient prognosis and survival. Subsequent experimental verification demonstrated that C5aR1 promoted the progression of GBM mainly by suppressing ferroptosis induction, inhibiting the accumulation of lipid peroxides, and stabilizing the expression of the core antiferroptotic factor glutathione peroxidase 4 (GPX4). Aberrant N6-methyladenosine (m6A) modification of GPX4 mRNA contributes significantly to epigenetic tumorigenesis, and here, we report that selective methyltransferase-like 3 (METTL3)-dependent m6A methylation of GPX4 plays a key role in C5AR1 knockdown-induced ferroptosis induction. Mechanistically, ERK1/2 signaling pathway activation increases the METTL3 protein abundance in GBM cells. This activation then increases the stability of METTL3-mediated m6A modifications on GPX4, enabling it to fulfill its transcriptional function. More importantly, in an intracranial xenograft mouse model, PMX205, a C5aR1 inhibitor, promoted alterations in ferroptosis in GBM cells and inhibited GBM progression. In conclusion, our findings suggest that C5aR1 inhibits ferroptosis in GBM cells and promotes MettL3-dependent GPX4 expression through ERK1/2, thereby promoting glioma progression. Our study reveals a novel mechanism by which the intracellular complement receptor C5aR1 suppresses ferroptosis induction and promotes GBM progression. These findings may facilitate the identification of a potential therapeutic target for glioma.

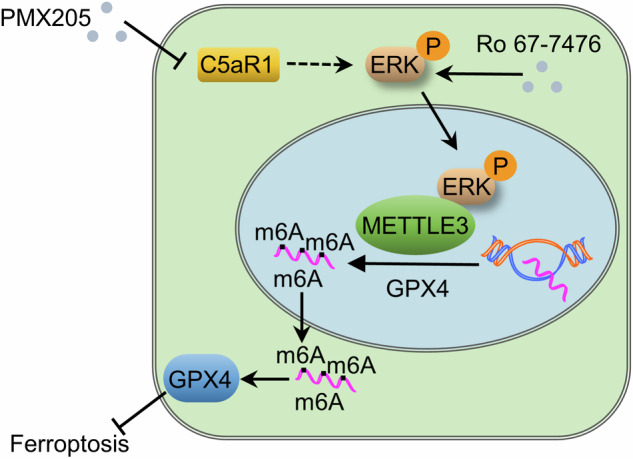

## Introduction

Glioblastoma (GBM) is the most common primary central nervous system malignancy in adults and accounts for approximately 49% of malignant brain tumors [1, 2], with the poor efficacy of current therapeutic strategies [3, 4]. Gliomas often exhibit heterogeneous lipid metabolic reprogramming [5], previous literature indicates that the deletion of a specific oncogene promotes increased lipid peroxidation in GBM cells and selectively triggers ferroptosis in tumors [5, 6]. Therefore, a comprehensive investigation of the mechanism of ferroptosis in GBM could help to identify key biomarkers and develop effective treatment strategies.

Ferroptosis is a new type of programmed cell death caused by iron-dependent lipid peroxidation and is completely distinct from other modes of cell death, such as apoptosis, necrosis, and autophagy [7]. Ferroptosis is characterized by the accumulation of intracellular lipid reactive oxygen species (ROS) and the conversion of intracellular lipid hydroperoxides (LOOHs) to toxic lipid free radicals (LO-) [8, 9]. The glutathione peroxidase 4 (GPX4) axis plays a key role in the regulation of ferroptosis through the conversion of reduced glutathione (GSH) to oxidized glutathione (GSSG) and reducing cytotoxic LOOHs to the corresponding alcohols (LOHs). Therefore, inhibiting GPX4 in tumors is a viable treatment strategy [10, 11]. Previous studies have shown that the synthetic small molecule GPX4 inhibitor RSL3 can effectively inhibit the development of colorectal cancer [12], breast cancer [13], and glioma [14]. However, its clinical application is limited by its undetermined toxicity and side effects [15]. Therefore, the development of new strategies to inhibit GPX4 has become a research focus.

During GBM development, various aberrations in epigenetic modifications, including DNA methylation, histone acetylation, and N6-methyladenosine (m6A) RNA modification, occur [16]. Among these modifications, m6A methylation is a common, abundant, and conserved internal transcriptional modification [17, 18]. m6A modification of RNA is dynamically regulated by several m6A methyltransferases, among which methyltransferase-like 3 (METTL3) and methyltransferase-like 14 (METTL14) are m6A “writers” that form the heterodimeric catalytic core and play a decisive role [19]. The initiation of ferroptosis in GBM cells is largely influenced by the m6A methylation of key ferroptosis genes [20, 21]. During the search for insights into epigenetic regulation, numerous sites of m6A enrichment, which is a marker of m6A methylation, were found in mature GPX4 mRNA [22]. m6A modification of GPX4 has been reported to occur under various ferroptotic conditions, but GPX4 modification mediated by METTL3 is less studied, especially in GBM [23–25]. These observations suggest that targeting GPX4 expression through interference with m6A modification is an effective strategy for inducing ferroptosis in GBM cells.

Complement C5a receptor 1 (C5aR1), the first-discovered major C5a receptor, is a typical G protein-coupled receptor. C5aR1 is expressed in a variety of cells, including myeloid-derived cells (such as macrophages, neutrophils, and monocyte subpopulations), specific lymphocytes, and tumor cells [26]. C5aR1 is highly expressed in various cancers, including lung, breast, and gastric cancers, and is associated with patient prognosis [27–29]. However, C5aR1 has not been studied as thoroughly in glioma. Previous studies have shown that C5aR1 can cause tumor-related immunity by inducing the production of bioactive molecules in the tumor microenvironment [30, 31]. Moreover, intracellular C5aR1 has been reported to interact with KCTD5/Cullin3/Roc1/β-catenin to form a stable complex, thereby increasing the stability of β-catenin and promoting the occurrence of colorectal cancer [32]. In general, targeting C5aR1 for tumor treatment has considerable potential utility, but the specific mechanism of action involving C5aR1 needs further exploration and study.

We thus investigated the key role and properties of intracellular C5aR1 in the progression of GBM. We focused on GPX4 expression and its potential epigenetic regulation by METTL3. Two models of GBM cells and an intracranial xenograft mouse model were used to study GBM cells ferroptosis induced by transfection of small interfering RNAs (siRNAs) targeting C5aR1 and treatment with PMX205 [33], a pharmacological inhibitor of C5aR1. Our research will contribute to a better understanding of the key epigenetic events regulating ferroptosis during GBM progression and provide insight into potential gene-targeting strategies to intervene in this process.

## Materials and methods

### Database analysis

The differential expression of C5aR1 between glioma tissues and adjacent noncancerous tissues was analyzed using the Cancer Genome Atlas (TCGA; https://cancergenome.nih.gov/) and the Chinese Glioma Genome Atlas (CGGA; http://www.cgga.org.cn/). Kaplan‒Meier survival analysis was then performed.

### Human glioma tissues

Glioma tissue sections (*n* = 36) were obtained from the Pathology Department of Yancheng Clinical College of Xuzhou Medical University (Yancheng, China) between January 1, 2020, and December 30, 2023. Normal brain samples (*n* = 8) were collected from patients with intracranial hypertension during craniotomy in the Department of Neurosurgery. All patients provided informed consent, and the clinical information related to the human samples is shown in Table 1. The specimens were acquired immediately after surgical resection, snap-frozen in liquid nitrogen, and stored at −80 °C. The study was approved by the Ethics Committee of Yancheng Clinical College of Xuzhou Medical University (no. 2023-K-088).

**Table 1:** Glioma characteristics of patients in C5aR1 low-expression and C5aR1 high-expression groups.

Feathers	Number	C5aR1 Low	C5aR1 High	*P* value
All patients	36	16	20	
Gender				0.709
Male	17	7	10	
Female	19	9	10	
Age at diagnosis				0.296
<55	16	6	10	
≥55	20	11	9	
Grade				0.012
I–II	10	6	4	
III	10	3	7	
IV	16	1	15	

### Cell culture and reagents

Human GBM cell lines (U87 and U251) and normal human astrocytes (HAs) were purchased from the American Type Culture Collection. U87, U251 and HAs were cultured in Dulbecco’s modified Eagle’s medium (C11995500BT, Gibco, USA) supplemented with 10% fetal bovine serum (16000-044, Gibco, USA), 100 µg/mL streptomycin and 100 U/mL penicillin (15140-122, Gibco, USA). The cells were cultured in an incubator at 37 °C with 5% CO_2_. All cell lines were authenticated via short tandem repeat profiling. The apoptosis inhibitor Z-VAD-FMK (HY-16658B, MedChemExpress MCE USA), the ferroptosis inhibitors ferrostatin-1 (HY-100579, MCE, USA) and liproxstatin-1 (HY-12726, MCE, USA), the necroptosis inhibitor necrostatin-1 (HY-15760, MCE, USA), the autophagy inhibitor 3-methyladenine (HY-19312, MCE, USA) and the C5aR antagonist PMX205 (HY-110136A, MCE, USA) were used. All the drugs were separately dissolved in dimethyl sulfoxide (DMSO; GC203002 Servicebio, China) or H_2_O according to the instructions.

### Cell counting kit-8 (CCK-8) assay

Cell viability was assessed with a CCK-8 (BMU106-CN, Abbkine, China). GBM cells were seeded into 96-well plates at a density of 3000 cells per well and incubated for 0 h, 24 h, 48 h, and 72 h in a controlled environment at 37 °C. Then, 10 µL of CCK-8 reagent was added to each well, and the plates were incubated for 2 h. The absorbance was measured at 450 nm with a multifunctional microplate reader (Thermo Fisher Scientific, USA). Cell viability was calculated as follows: [(experimental well absorbance − blank well absorbance)/(reference well absorbance − blank well absorbance)] × 100%.

### Calcein AM/propidium iodide (PI) staining

Calcein AM/PI staining was performed using a Calcein AM/PI double staining kit (E-CK-A354, Elabscience, China) according to the manufacturer’s protocol. In brief, cells were washed twice with PBS and incubated with calcein AM solution and PI solution for 20 min. Images were then acquired using a fluorescence microscope (Olympus, Tokyo, Japan).

### Quantitative reverse transcription PCR (RT-qPCR)

Total RNA was isolated from cells or tumor tissues using an RNA-easy Isolation Reagent (R701-01, Vazyme, China). Equal amounts of mRNA were reverse transcribed to cDNA with HiScript II Q RT SuperMix for qPCR (+gDNA Wiper) (R223, Vazyme, China). ChamQ Universal SYBR qPCR Master Mix (Q711 Vazyme) was mixed with the cDNA and gene-specific primers, and qPCR was performed according to the manufacturer’s protocol. The GPX4 mRNA level was determined by normalization to the corresponding β-actin level using the 2^−∆∆Ct^ method. The primers used were as follows: GPX4 forward, 5’-GAGGCAAGACCGAAGTAAACTAC-3’; GPX4 reverse, 5’- CCGAACTGGTTACACGGGAA-3’; β-actin forward, 5’-CTCCATCCTGGCCTCGCTGT-3’; β-actin reverse, 5’-GCTGTCACCTTCACC GTTCC-3’.

### Western blotting

Total protein was extracted using radioimmunoprecipitation assay buffer (G2002, Servicebio, China) supplemented with a phosphatase and protease inhibitor (P1005, Beyotime, China). The protein concentration was measured using a bicinchoninic acid (BCA) kit (23225, Thermo Fisher Scientific, USA), and equal amounts of protein were loaded onto a sodium dodecyl sulfate (SDS)–polyacrylamide gel and transferred to a polyvinylidene fluoride membrane (Millipore, USA). Following overnight incubation with the primary antibody at 4 °C, the membrane was further incubated with horseradish peroxidase-conjugated secondary antibody and enhanced chemiluminescence solution (BMU101-CN, Abbkine, China). A ChemiDoc imaging system (Bio-Rad, USA) was used for visualization. Densitometric quantification was performed using β-actin as the control. The following primary antibodies were used: anti-C5aR (21316-1-AP, Proteintech, China), anti-GAPDH (10494-1-AP, Proteintech, China), anti-GPX4 (ab125066, Abcam, USA), anti-4-hydroxynonenal (4-HNE; ab46545, Abcam, USA), anti-Flag (66008-4-Ig, Proteintech, China), anti-METTL3 (67733-1-Ig, Proteintech, China), anti-METTL14 (26158-1-AP, Proteintech, China), anti-HA (51064-2-AP, Proteintech, China), anti-ERK1/2 (11257-1-AP, Proteintech, China), and anti-p-ERK1/2 (28733-1-AP, Proteintech, China).

### Immunofluorescence (IF) staining

Cells were seeded onto glass slides and fixed with 4% paraformaldehyde. Then, the cells were permeabilized using Triton X-100 and incubated overnight at 4 °C with primary antibodies. Afterward, the samples were incubated with Alexa Fluor 594-conjugated donkey anti-mouse IgG (A24411, Abbkine, China) or IFKine™ Green Donkey Anti-Mouse IgG (A24211, Abbkine, China). Nuclei were stained using 4’,6-diamidino-2-phenylindole (DAPI; BMU107-CN, Abbkine, China), and the cell membrane was stained using DiO (DiOC18(3), BMD0072, Abbkine, China). IF was evaluated using a fluorescence microscope (Hitachi HT7700, Tokyo, Japan).

### Transmission electron microscopy (TEM)

Cell samples were collected using low-speed centrifugation and fixed in an electron fixation solution containing 2.5% glutaraldehyde (G1102, Servicebio, China). After postfixation with 1% osmium tetroxide and dehydration, the samples were embedded in Epon. The sections were stained with uranyl acetate and examined using a transmission electron microscope (Hitachi HT7700, Japan).

### Malondialdehyde (MDA) assay

The content of the lipid peroxidation product MDA was measured according to the manufacturer’s instructions (KTB1050, Abbkine, China). In brief, approximately 5 × 10^6^ cells from each sample were incubated with the reaction mixture at 95 °C for 30 min. To measure the MDA and BCA contents, the absorbance was measured using a multifunctional microplate reader (Thermo Fisher Scientific, USA). The MDA content was determined based on the standard curve.

### GSH assay

The GSH content in GBM cells was measured following the manufacturer’s instructions (KTB1600, Abbkine, China). In brief, approximately 2 × 10^6^ cells from each sample were incubated with the reaction mixture at room temperature for 2 min in the dark. To measure the GSH content, the absorbance was measured using a multifunctional microplate reader (Thermo Fisher Scientific, USA).

### Intracellular ROS detection

For ROS detection, cells were treated with 2.5 μmol/L 2,7-dichlorodihydrofluorescein diacetate (DCFH-DA; D6883-50MG, Sigma-Aldrich, USA) for 1 h at 37 °C. Oxidation of intracellular fluorophores was then detected by flow cytometry using an excitation wavelength of 488 nm and an emission wavelength of 535 nm. The results are expressed as the mean fluorescence intensity values.

### C11-BODIPY 581/591 staining

GBM cells were stained with 10 μM C11-BODIPY 581/591 at 37 °C for 1 h in the dark and washed three times with PBS. Nuclei were then stained with DAPI solution in PBS at 37 °C for 10 min in the dark and washed three times with PBS. Green fluorescence indicates oxidized lipids, while red fluorescence indicates nonoxidized lipids. The increase in green fluorescence and decrease in red fluorescence reflects the ratio of oxidized to nonoxidized lipids. The images were analyzed using ImageJ.

### Intracranial xenograft model establishment and imaging

The Animal Care Committee of Jiangsu Vocational College of Medicine approved all the animal experiments (Approval XMLL-2023-746). To establish the intracranial xenograft model, we injected 1 × 10^6^ U87 cells engineered to express luciferase into the right striatum of experimental nude mice (6 weeks old) after anesthetizing them via isoflurane inhalation. The cells were injected 3.5 mm lateral to the midline of the brain and 2 mm anterior to the coronal suture at a depth of 3 mm from the brain surface. The mice were randomly divided into two groups (*n* = 6 mice per group) and treated with PBS or PMX205 (5 mg/kg) every two days for four weeks beginning on day 7 after tumor cell inoculation. Additionally, we monitored tumor progression by imaging the intracranial tumors with an in vivo imaging system (IVIS) at 1 week, 3 weeks, and 5 weeks after tumor cell inoculation. The mice were monitored regularly and euthanized when they exhibited severe neurological symptoms or weight loss exceeding 20% of their initial body weight. After the experiment was completed, the brains of the mice were surgically removed, fixed, embedded, and sectioned for immunohistochemical (IHC) staining.

### IHC staining

IHC staining was used to evaluate the level and distribution of proteins in tumor tissues. In brief, tumors were fixed with 4% paraformaldehyde and embedded in paraffin. The tumors were then sectioned, deparaffinized, hydrated, and washed. The sections were incubated with a primary antibody specific for GPX4 or 4-HNE overnight at 4 °C. The next day, the sections were washed and incubated with secondary antibodies. Immunoreactive signals were detected with 3,3’-diaminobenzidine. Nuclei were counterstained with hematoxylin.

### Plasmids, RNA interference, and transfection

The GPX4 (NM_002085) overexpression plasmid was constructed and supplied by GeneChem Co. (Shanghai, China). In brief, GPX4 cDNA was amplified and inserted into the CMV enhancer-MCS-polyA-EF1A-sv40-puromycin vector with a Flag tag. The METTL3 (NM_019721) overexpression plasmid was purchased from YouBio (Hunan, China). In brief, METTL3 cDNA was amplified and inserted into the pLVX-TetOne-Puro vector with an HA tag. The pLVX-TetOne-Puro vector was a gift from Rongqing Li (Xuzhou Medical University, Xuzhou, China).

Specific siRNAs targeting C5aR1 were purchased from GenePharma (Shanghai, China). The sequences of the siRNAs were as follows: si-C5AR1-homo1#: sense, CCCUCAUCCUGCUCAACAUTT and antisense, AUGUUGAGCAGGAUGAGGGTT; si-C5AR1-homo2#: sense, GGACUACAGCCACGACAAATT and antisense, UUUGUCGUGGCUGUAGUCCTT. si-METTL3-homo1#: sense, UGUUUAUUGAUAAUUCGUCUG and antisense, GACGAAUUAUCAAUAAACACA; si- METTL3-homo2#: sense, UGUGUUUAUUGAUAAUUCGUC and antisense, CGAAUUAUCAAUAAACACACU.

GBM cells were transfected with the indicated plasmids and siRNAs using jetPRIME transfection reagent (0000001762, Polyplus, France) according to the manufacturer’s instructions.

### Dot blot

Total RNA was isolated by Trizol Reagent (R701-01, Vazyme, China) and then mixed with a triploid RNA incubation buffer formulated with formamide, methanol, and 10× MOPS. The RNA samples were degenerated at 65 °C for 5 minutes and then cooled on ice. The sample was mixed with the same volume of 20× SSC, soaked with water, and washed with 10× SSC. Ultraviolet lamp treatment for 30 min, PBST wash three times. The membrane was then blocked with 5% skim milk and treated with anti-m6A antibodies (1:2000; 68055-1-Ig, Proteintech, USA) and anti-mouse antibodies (1:2000; SA00001-1, Proteintech, USA). A ChemiDoc imaging system (Bio-Rad, USA) was used for visualization.

### Methylated RNA immunoprecipitation (MeRIP)–PCR

Total RNA was extracted from GBM cells subjected to different treatments and processed with a Dynabeads™ mRNA Purification Kit (No. 61006; Invitrogen, USA) to remove genomic DNA. After mRNA purification and fragmentation, the fragments were incubated with an anti-m6A primary antibody for immunoprecipitation via a Magna MeRIP™ m6A Kit (A-P-9018, IVDSHOW, China). Enriched m6A-modified mRNAs were then analyzed via RT-qPCR. The primers used were as follows: GPX4 Primer 1 Forward, CCTTTGCCGCCTAC.

TGAAG; GPX4 Primer 1 reverse, GTCGACGAGCTGAGTGTAGT; GPX4 Primer 2 forward, CCTTGGAGCCTTCCACCG; GPX4 Primer 2 reverse, CACAAGGTAGCCAGGGGTG.

### Coimmunoprecipitation (Co-IP) assay

Total protein was extracted from GBM cells using NP-40 cell lysis buffer (P0013F; Beyotime, China) supplemented with protease inhibitors. After centrifugation, the proteins in the supernatant were separated by SDS-PAGE and were then analyzed by immunoblotting. The cell lysates were incubated overnight at 4 °C with an anti-p-ERK1/2 antibody (4 μg/mL) or control IgG (1:100; AC011, ABclonal, China) and were then incubated with protein A/G beads (20241, Invitrogen, USA) for 3 h at 4 °C. The beads were carefully washed with precooled PBS, mixed with protein loading buffer, boiled, and analyzed by immunoblotting using an anti-METTL3 antibody (1:500) and an anti-p-ERK1/2 antibody (1:500).

### Statistical analysis

To compare continuous variables between two groups and among more than two groups, unpaired Student’s *t*-test and one-way or two-way ANOVA, respectively, were employed. The results are presented as the means, and the error bars indicate the standard deviations. We performed statistical analyses using GraphPad Prism 8.0. Bioinformatic analyses were performed as previously described. The criteria for statistical significance were established as follows: *P < 0.05; **P < 0.01, ***P < 0.01; ns, nonsignificant.

## Results

### C5aR1 is upregulated and associated with prognosis in GBM

**Fig. 1:**
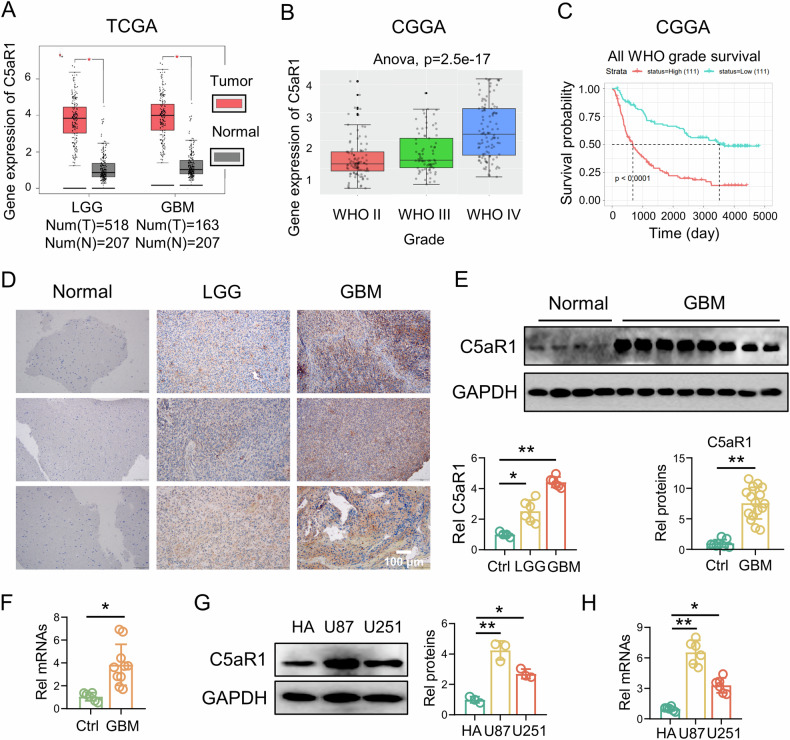
C5aR1 is upregulated and associated with prognosis in GBM. **A** A TCGA dataset was used to analyze the expression of C5aR1 in GBM tissue (*n* = 163), LGG tissue (*n* = 518), and nontumor brain tissue (*n* = 207); *P < 0.05. **B** A CGGA dataset was used to analyze the expression of C5aR1 in grade II, grade III, and grade IV glioma tissues and nontumor brain tissues; ANOVA, *P* = 2.5e–17. **C** Kaplan–Meier survival analysis of clinically diagnosed glioma patients based on C5aR1 expression was performed with a CGGA dataset; *P* < 0.0001. **D** IHC staining of C5aR1 in LGG (*n* = 6), GBM (*n* = 6), and normal brain tissues (*n* = 4) and the quantitative analysis, (means ± SD, *P < 0.05, **P < 0.01). **E** C5aR1 protein expression levels in GBM tissues (*n* = 16) and normal brain tissues (*n* = 8) from different patients were determined by western blotting and quantitatively analyzed. The data are presented as the means ± SD. **P < 0.01. **F** Differential mRNA expression of C5aR1 in GBM tissues (*n* = 10) and normal brain tissues (*n* = 6), as measured by RT-qPCR. The data are presented as the means ± SD. *P < 0.05. **G** C5aR1 protein expression in HA, U87, and U251 cells was detected by Western blotting and quantitatively analyzed, (*n* = 3, means ± SD, *P < 0.05, **P < 0.01). **H** Differential mRNA expression of C5aR1 among HA, U87, and U251 cells was analyzed by RT-qPCR, (*n* = 6, means ± SD, *P < 0.05, **P < 0.01).

To investigate the role of C5aR1 in glioma pathogenesis, we examined its expression in glioma tissue using data from the TCGA database and the CGGA. According to the analysis of TCGA data, C5aR1 was significantly upregulated in low-grade glioma (LGG) tissue (*n* = 518) and GBM tissue (*n* = 163) compared to normal brain tissue (*n* = 207). Analysis of CGGA data showed that C5aR1 expression increased with increasing glioma grade and that C5aR1 overexpression was associated with poor prognosis in patients with glioma, as indicated by the low survival rate of patients with high C5aR1 expression (Fig. 1A–C). In addition, we performed IHC analysis of normal brain tissue, LGG tissue, and GBM tissue from patients. The results confirmed that C5aR1 was upregulated specifically in glioma tissue, especially in GBM tissue (Fig. 1D). We further divided the glioma patients from Yancheng Clinical College of Xuzhou Medical University into the C5aR1 high- and low-expression groups and analyzed the correlations between the C5aR1 expression level and the clinicopathological parameters of the glioma patients. WHO classification was significantly greater in the high-expression group than in the low-expression group (Table 1). Furthermore, analysis of RNA and protein expression in both normal brain tissue and GBM tissue further supported the finding that C5aR1 was more highly expressed in GBM tissue than in normal brain tissue (Fig. 1E, F). To further investigate the expression of C5aR1 in various cell lines, RNA and protein were extracted from a normal human astrocyte cell line (HA) and two GBM cell lines (U87 and U251). The expression level of C5aR1 was higher in both U87 and U251 cells than in HA cells (Fig. 1G, H).

### Knockdown of intracellular C5aR1 selectively triggers regulated ferroptotic cell death in GBM cells

**Fig. 2:**
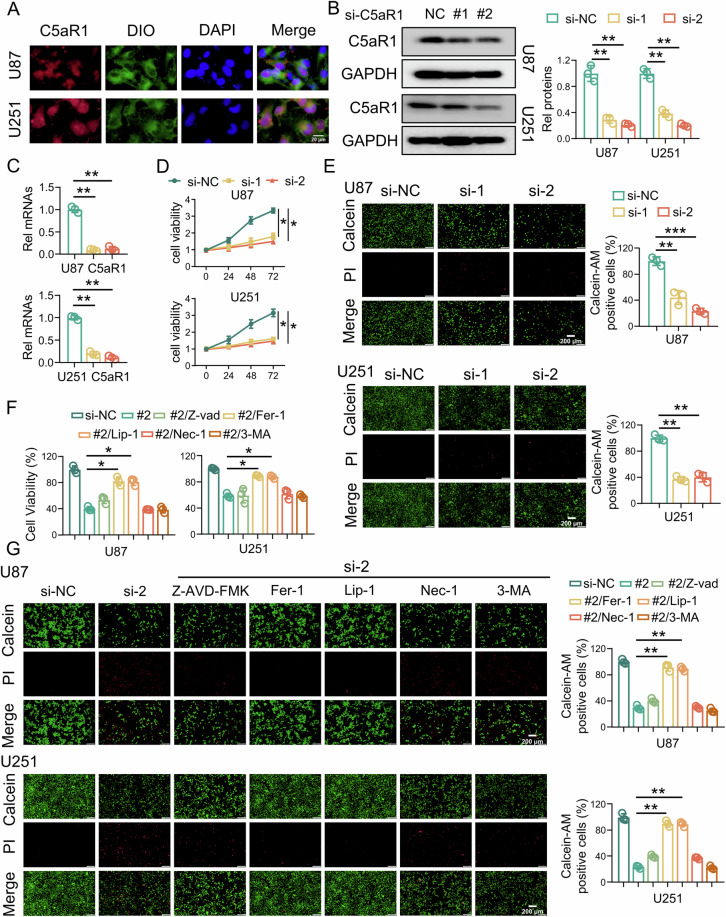
Knockdown of intracellular C5aR1 selectively triggers regulated ferroptotic cell death in GBM cells. **A** IF staining of U87 and U251 cells with an anti-C5aR1 antibody (red) and a cell membrane dye (green). C5aR1 knockdown decreased the intracellular C5aR1 level in GBM cells. Scale bar, 20 μm. **B** After U87 and U251 cells were transfected with siRNA, Western blotting was used to measure the protein expression of C5aR1, and quantitative analysis was performed, (*n* = 3, means ± SD, **P < 0.01). **C** RT-qPCR was used to verify the transfection efficiency of si-C5aR1, (*n* = 3, means ± SD, **P < 0.01). **D** A CCK-8 assay was used to evaluate changes in GBM cell viability after C5aR1 knockdown, (*n* = 3, means ± SD, *P < 0.05). **E** Calcein AM/PI staining of U87/U251 cells after C5aR1 knockdown was detected via fluorescence microscopy, (*n* = 3, means ± SD, **P < 0.01, ***P < 0.001). Scale bar, 200 μm. **F** A CCK-8 assay was used to evaluate changes in the viability of GBM cells treated with or without Z-VAD-FMK (Z-VAD-FMK, 20 μM), ferrostatin-1 (Fer-1, 10 μM), liproxstatin-1 (Lip-1, 20 μM), necrostatin-1 (Nec-1, 10 μM), or 3-methyladenine (3-MA, 500 μM) for 48 h after C5aR1 knockdown, (*n* = 3, means ± SD, *P < 0.05). **G** Calcein AM/PI staining was detected via fluorescence microscopy in U87/U251 cells treated with or without Z-VAD-FMK, Fer-1, Lip-1, Nec-1, or 3-MA for 48 h after C5aR1 knockdown, (*n* = 3, means ± SD, **P < 0.01).

To further explore the potential role of C5aR1 in GBM cells, further analysis was performed by IF staining. Unexpectedly, C5aR1 was detected not only on the cell membrane but also in the cytoplasm of GBM cells (Fig. 2A). In order to elucidate the mechanism of action of C5aR1 in GBM cells, C5a was detected using IF staining. The findings indicate the presence of C5a in GBM cells, which suggests that C5aR1 in GBM cells is mainly activated by C5a to promote cancer (Fig. S1). To examine the impact of C5aR1 on GBM cells, siRNA was used to knock down C5aR1 in U87 and U251 cells. The knockdown efficiency was validated through RT-qPCR and western blotting (Fig. 2B, C). Subsequently, a significant reduction in the intracellular C5aR1 level in U87 and U251 cells was observed after C5aR1 knockdown (Fig. S2). The results of the CCK-8 cell viability assay demonstrated that knockdown C5aR1 decreased the viability of GBM cells (Fig. 2D). Furthermore, calcein AM/PI staining revealed a substantial decrease in the number of living cells and an increase in the number of dead cells among C5AR1 knockdown GBM cells compared to control cells (Fig. 2E). To explore the specific mechanism by which C5aR1 knockdown induces GBM cell death, several inhibitors were used to treat GBM cells to clarify the role of C5aR1 in regulating cell death [34]. The CCK-8 assay and calcein AM/PI staining [35] revealed that the classical apoptosis inhibitor Z-VAD-FMK, the necroptosis inhibitor necrostatin-1, and the autophagy inhibitor 3-methyladenine did not significantly reverse the effects of C5aR1 knockdown. However, the ferroptosis inhibitors ferrostatin-1 and liproxstatin-1 significantly reversed the effect of C5aR1 knockdown on inducing cell death (Fig. 2F, G).

### Knockdown of intracellular C5aR1 increases lipid peroxidation and induces ferroptosis via suppression of GPX4 expression in GBM cells

**Fig. 3:**
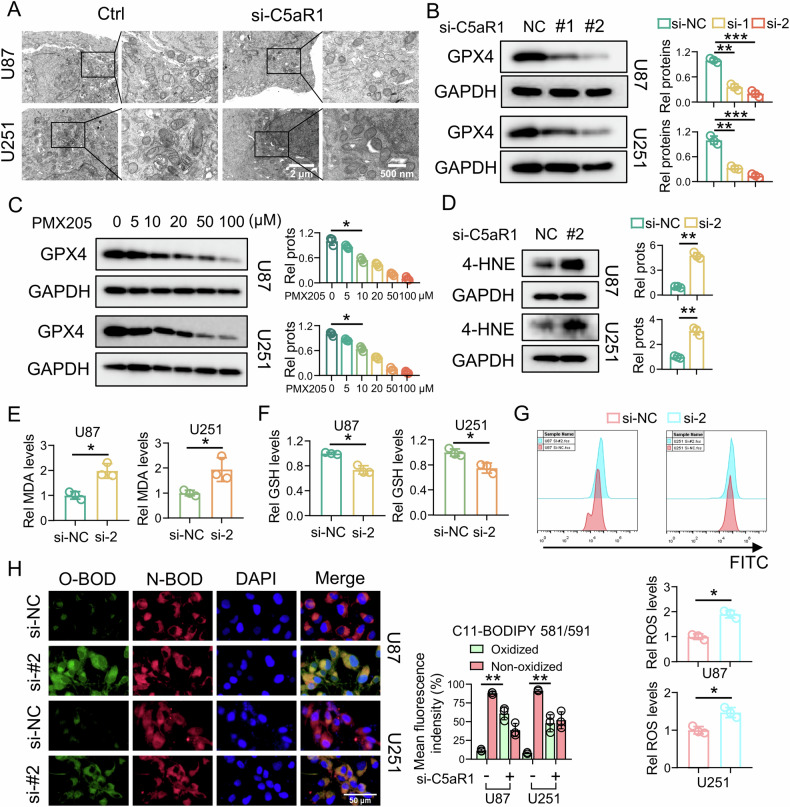
Knockdown of intracellular C5aR1 increases lipid peroxidation and induces ferroptosis via suppression of GPX4 expression in GBM cells. **A** TEM was used to visualize ferroptosis in U87 and U251 cells. Scale bars, 2 μm, and 500 nm. **B** Protein expression of GPX4 after C5aR1 knockdown, and the related quantitative analysis, (*n* = 3, means ± SD, **P < 0.01, ***P < 0.001). **C** Protein expression of GPX4 after treatment with different concentrations of PMX205, and the related quantitative analysis, (*n* = 3, means ± SD, *P < 0.05). **D** Abundance of 4-HNE after C5aR1 knockdown, and the related quantitative analysis, (*n* = 3, means ± SD, **P < 0.01). **E** Intracellular MDA levels after C5aR1 knockdown, and the related quantitative analysis, (*n* = 3, means ± SD, *P < 0.05). **F** GSH level after C5aR1 knockdown, and the related quantitative analysis, (*n* = 3, means ± SD, *P < 0.05). **G** Relative ROS levels after C5aR1 knockdown, and the related quantitative analysis, (*n* = 3, means ± SD, *P < 0.05). **H** Oxidation-induced fluorescence of C11-BODIPY 581/591 in U87/U251 cells transfected with si-C5aR1. Green fluorescence indicates the oxidation reaction (O-BOD), while red fluorescence indicates the reduction reaction (N-BOD) The related quantitative analysis is shown, (*n* = 3, means ± SD, **P < 0.01). Scale bar, 50 μm.

Previous studies found that the most classical manifestations of ferroptosis are that mitochondria shrink, the mitochondrial membrane density increases, the cristae decrease in height and even disappear, and the outer membrane ruptures [36]. To determine whether the death of glioma cells induced by C5aR1 knockdown is due primarily to ferroptosis, we performed TEM, which showed that C5aR1 knockdown caused extensive changes in mitochondria, such as mitochondrial shrinkage, loss of cristae, and rupture of the outer membrane (Fig. 3A). In investigating the cause of ferroptosis, we discovered that knockdown of C5aR1 led to decreases in protein levels of GPX4, an antiferroptotic enzyme that catalyzes the reduction of lipid peroxides. Furthermore, we observed that the C5aR1 inhibitor PMX205 dose-dependently reduced the protein abundance of GPX4 (Fig. 3B, C). In addition, we conducted TEM to observe the extensive changes in the mitochondria of U87 cells after PMX205 treatment, such as mitochondrial contraction, ridge loss, and outer membrane rupture (Fig. S3). The accumulation of lipid peroxides is also a characteristic of ferroptosis. To demonstrate that C5aR1 inhibits ferroptosis by targeting GPX4, we measured the levels of the lipid peroxidation markers 4-HNE and MDA. Knockdown of C5aR1 clearly caused the accumulation of 4-HNE and MDA (Fig. 3D, E). GSH can scavenge ROS in cells via a reaction catalyzed by GPX4 [9]. Therefore, we measured the levels of intracellular GSH and ROS in GBM cells with C5aR1 knockdown. The results showed that GSH depletion (Fig. 3F) and ROS accumulation (Fig. 3G) were significantly increased following C5aR1 knockdown. To validate these results, we treated GBM cells with C11-BODIPY (boron dipyrromethane difluoride), a fatty acid analog that emits red fluorescence that shifts to green fluorescence upon oxidation, and found that C5aR1 knockdown caused a shift from red to green fluorescence (Fig. 3H). In addition, to explore whether C5aR1 knockdown can increase the effect of classical ferroptosis inducers, we used C5aR1 knockdown and classical GPX4 inhibitor RSL3. The unexpected findings indicated that the downregulation of C5aR1 in U87 cells notably augmented the antitumor efficacy of RLS3, whereas this effect was not observed in U251 cells. These results imply that the interaction between distinct cell lines may yield varying outcomes (Fig. S4).

### The C5aR1 inhibitor PMX205 can induce ferroptosis in GBM cells in an intracranial xenograft model

**Fig. 4:**
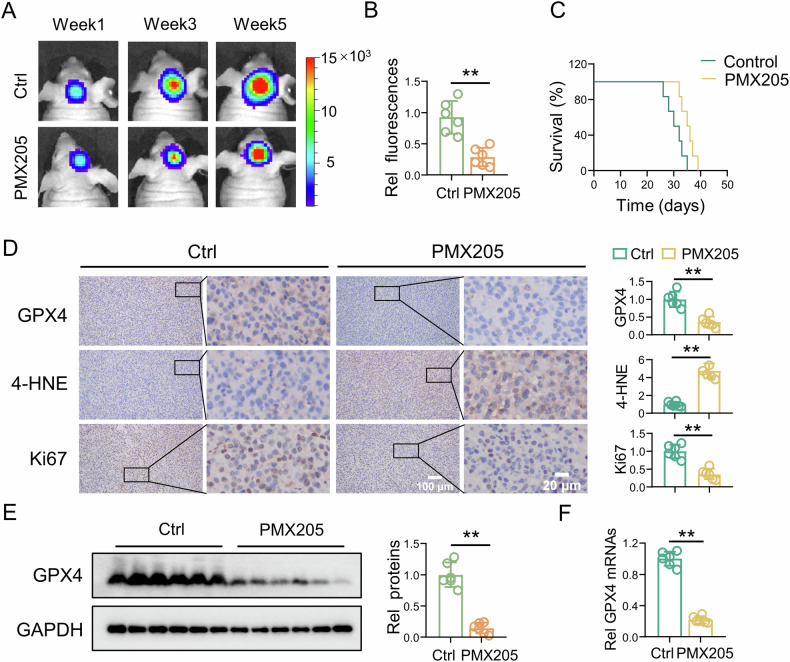
The C5aR1 inhibitor PMX205 can induce ferroptosis in GBM cells in an intracranial xenograft model. **A** Representative bioluminescence images showing luciferase signals in tumors in mice in the different treatment groups at 1 week, 3 weeks, and 5 weeks acquired by IVIS imaging. **B** Quantitative analysis of the data in (**A**). The data are presented as the means ± SD, (*n* = 6, **P < 0.01). **C** Mouse survival is shown on Kaplan–Meier curves (*n* = 6). The *P* value was calculated using the log-rank test. *P* = 0.0141. **D** IHC staining of GPX4, 4-HNE, and Ki67 in tumor tissue, and the related quantitative analysis, (*n* = 6, means ± SD, **P < 0.01). Scale bars, 100 μm and 20 μm. **E** Protein expression of GPX4 after the indicated treatment, and the related quantitative analysis, (*n* = 6, means ± SD, **P < 0.01). **F** Differential mRNA expression of GPX4 in tumor tissues, as measured by RT-qPCR, (*n* = 6, means ± SD, **P < 0.01).

To assess the effect of C5aR1 on GBM in vivo, we established an intracranial xenograft GBM model. U87 cells were transduced with lentiviral vectors for stable expression of luciferase, and the xenograft tumors formed from these cells were evaluated with an IVIS. Compared with the control treatment, PMX205 treatment significantly reduced the tumor size (Fig. 4A, B). Survival analysis revealed that PMX205 prolonged the survival of control mice (Fig. 4C). Furthermore, IHC staining confirmed that PMX205 induced suppression of GPX4 expression, accumulation of 4-HNE, and inhibition of tumor growth (Fig. 4D). PMX205 induced significant decreases in GPX4 mRNA and protein expression (Fig. 4E, F). To confirm if PMX205 induces ferroptosis in GBM in vivo, mice with intracranial xenograft tumors were treated with liproxstatin-1. Tumor size, survival time of mice, GPX4 protein levels, 4-HNE levels, and Ki67 expression were measured (Fig. S5). These results demonstrated that PMX205 induced ferroptosis and inhibited GBM progression in vivo.

### GPX4 overexpression blocks C5aR1 knockdown-induced ferroptosis alterations and lipid peroxidation in GBM cells

**Fig. 5:**
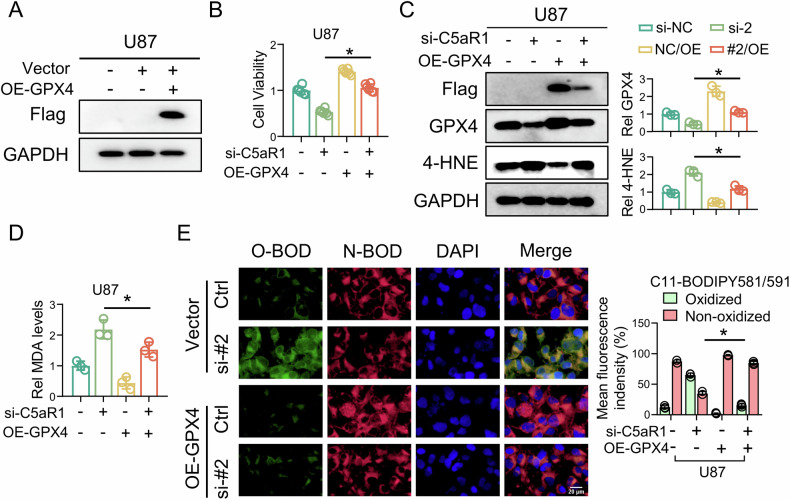
GPX4 overexpression blocks C5aR1 knockdown-induced ferroptosis alterations and lipid peroxidation in GBM cells. **A** Western blotting was used to detect the OE-GPX4 construct in U87 cells. **B** A CCK-8 assay was used to evaluate changes in the viability of cells treated with si-C5aR1 and/or OE-GPX4, (*n* = 3, means ± SD, *P < 0.05). **C** Western blotting was used to measure the protein expression of Flag, GPX4, and 4-HNE in cells transfected with si-C5aR1 and/or transduced with OE-GPX4, and the results were quantitatively analyzed, (*n* = 3, means ± SD, *P < 0.05). **D** Intracellular MDA level in cells transfected with si-C5aR1 and/or transduced with OE-GPX4, and the related quantitative analysis, (*n* = 3, means ± SD, *P < 0.05). **E** Oxidation-induced fluorescence of C11-BODIPY 581/591 in U87 cells transfected with si-C5aR1 and/or transduced with OE-GPX4, (*n* = 3, means ± SD, *P < 0.05). Scale bar, 20 μm.

To investigate the role of C5aR1 in regulating GPX4 expression to affect ferroptosis induction in GBM cells, a plasmid capable of overexpressing flag-tagged GPX4 (OE-GPX4) in U87 cells was constructed (Fig. 5A). GPX4 overexpression significantly attenuated the decrease in U87 cell viability (Fig. 5B), suppression of GPX4 expression, accumulation of 4-HNE (Fig. 5C), production of MDA (Fig. 5D) and accumulation of lipid peroxides induced by C5aR1 knockdown, as demonstrated by C11-BODIPY 581/591 staining (Fig. 5E), suggesting that suppression of GPX4 expression by C5aR1 knockdown is critical for the ferroptosis-inducing effects of C5aR1 knockdown.

### C5aR1 knockdown decreases METTL3-mediated m6A modification of GPX4

**Fig. 6:**
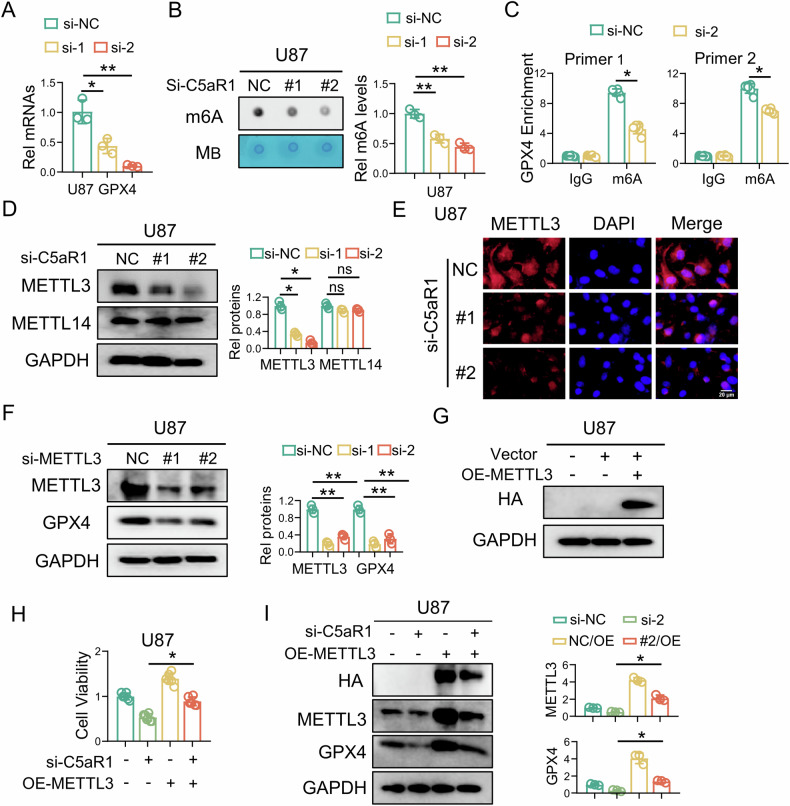
C5aR1 knockdown decreases METTL3-mediated m6A modification of GPX4. **A** Differential mRNA expression of GPX4 after transfection with si-C5aR1, as measured by RT-qPCR, (*n* = 3, means ± SD, *P < 0.05, **P < 0.01). **B** m6A dot blotting assays were used to measure m6A methylation levels after transfection with si-C5aR1 in U87 cells, and the results were quantitatively analyzed, (*n* = 3, means ± SD, **P < 0.01). **C** Differential m6A methylation of GPX4 after transfection with si-C5aR1, as measured by MeRIP, (*n* = 3, means ± SD, *P < 0.05). **D** Western blotting was used to measure the protein expression of METTL3 and METTL14 in cells transfected with si-C5aR1, and the results were quantitatively analyzed, (*n* = 3, means ± SD; *P < 0.05; ns, nonsignificant). **E** IF staining of U87 cells with an antibody against METTL3 (red). Scale bar, 20 μm. **F** Western blotting was used to measure the protein expression of METTL3 and GPX4 in cells transfected with si-METTL3, and quantitative analysis was performed, (*n* = 3, means ± SD, **P < 0.01). **G** Western blotting was used to detect the constructed OE-METTL3 plasmid in U87 cells. **H** A CCK-8 assay was used to evaluate the changes in GBM cell viability induced by transfection of si-C5aR1 and/or transduction of OE-METTL3, (*n* = 3, means ± SD, *P < 0.05). **I** Western blotting was used to measure the protein expression of HA, METTL3, and GPX4 in cells transfected with si-C5aR1 and/or transduced with OE-METTL3, and the results were quantitatively analyzed, (*n* = 3, means ± SD, *P < 0.05).

To investigate the regulation of GPX4 expression by C5aR1 in GBM cells, the GPX4 mRNA level was measured using RT-qPCR. C5aR1 knockdown significantly decreased the mRNA level of GPX4 (Fig. 6A). Previous studies have shown that GPX4 expression is regulated by epigenetic mechanisms [37]. To investigate the potential regulation of GPX4 transcriptional inhibition, we analyzed the expression of mature GPX4 mRNA using the online software SRAMP (https://www.cuilab.cn/sramp), and numerous m6A sites were found in the mature mRNA of GPX4. To determine m6A methylation levels after C5aR1 knockdown, a dot blot assay was used to detect in U87 cells. The results showed that m6A modification decreased after C5aR1 knockdown (Fig. 6B). Then we selected two very-high-confidence m6A sites of GPX4, to further confirm whether C5aR1 can mediate the m6A level of GPX4, we designed specific primers targeting the very-high-confidence region and performed MeRIP-qPCR [22]. C5aR1 knockdown significantly reduced the m6A level of GPX4 (Fig. 6C). Previous studies found that METTL3 and METTL14 are the primary methyltransferases responsible for m6A modification and play a crucial role in the formation of heterocomplexes [19]. To investigate the impact of C5aR1 on the GPX4 m6A level, we conducted western blotting to analyze the expression of METTL3 and METTL14. Surprisingly, the METTL3 level decreased significantly after C5aR1 knockdown, while the METTL14 level did not change significantly. Therefore, we speculated that C5aR1 regulates the GPX4 m6A level mainly through METTL3 (Fig. 6D). Further IF assays confirmed that C5aR1 knockdown decreased the expression of METTL3 in the nucleus (Fig. 6E). In addition, siRNA was designed to demonstrate the effect of METTL3 knockdown on GPX4 expression in GBM cells [38]. Western blotting revealed that si-METTL3 significantly inhibited GPX4 expression (Fig. 6F). TEM showed that si-METTL3 induced ferroptosis in the mitochondria of U87 cells (Fig. S6). To investigate the role of C5aR1 in regulating METTL3-dependent GPX4 m6A methylation in GBM cells, a plasmid capable of overexpressing METTL3 (OE-METTL3) in U87 cells was constructed (Fig. 6G). The experimental results indicated that METTL3 overexpression significantly attenuated the C5aR1 knockdown-induced reduction in U87 cell viability (Fig. 6H) and suppression of METTL3 and GPX4 expression (Fig. 6I).

### C5aR1 affects the METTL3 protein level by regulating the ERK1/2 pathway

**Fig. 7:**
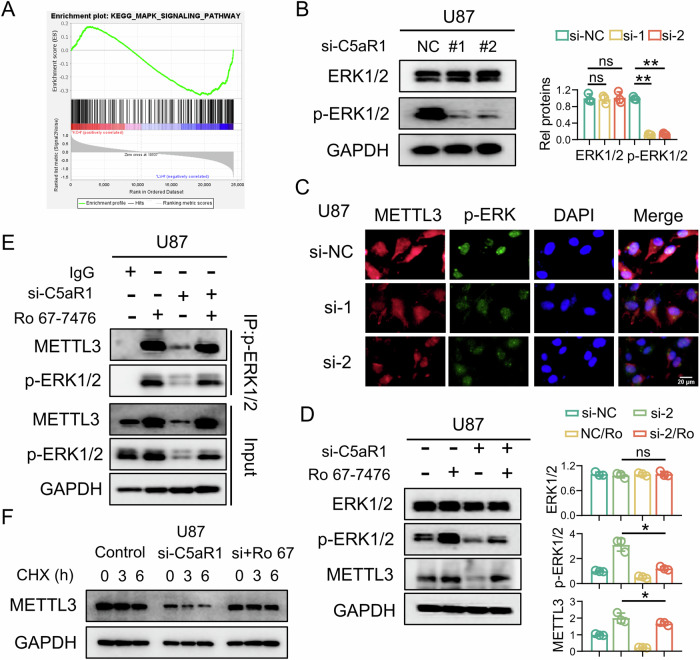
C5aR1 affects METTL3 by regulating the ERK1/2 pathway. **A** Gene set enrichment analysis showed enrichment of genes related to the MAPK-ERK1/2 pathway in U87 cells. **B** Western blotting was used to measure the protein levels of ERK1/2 and p-ERK1/2, and the results were quantitatively analyzed, (*n* = 3, means ± SD; **P < 0.01; ns, nonsignificant). **C** IF staining of U87 cells with antibodies against METTL3 (red) and p-ERK1/2 (green). Scale bar, 20 μm. **D** Western blotting was used to measure the protein levels of ERK1/2, p-ERK1/2, and METTL3 in cells transfected with si-C5aR1 and/or treated with Ro 67-7476, and quantitative analysis was performed, (*n* = 3, means ± SD; *P < 0.05; ns, nonsignificant. **E** Co-IP confirmed that p-ERK1/2 binds to METTL3 in U87 cells transfected with si-C5aR1 and/or treated with Ro 67-7476. **F** A CHX (50 μM) chase assay was performed to evaluate METTL3 protein stability in U87 cells transfected with si-C5aR1 and/or treated with Ro 67-7476.

Herein, we investigated the underlying mechanism by which C5aR1 modulates METTL3-mediated GPX4 m6A modification. Analysis of the CGGA-325 dataset revealed a significant correlation between C5aR1 expression and ERK1/2 pathway activity in gliomas (Fig. 7A). Further western blot analysis demonstrated that C5aR1 knockdown significantly reduced the levels of Thr202- and Tyr204-phosphorylated ERK1/2 (Fig. 7B). In addition, IF staining confirmed that p-ERK1/2 and METTL3 were colocalized in the nucleus and that C5aR1 knockdown significantly reduced the abundances of p-ERK1/2 and METTL3 in the nucleus (Fig. 7C). To confirm that C5aR1 regulates METTL3 expression by modulating p-ERK1/2, we treated U87 cells with Ro 67-7476, a potent p-ERK1/2 activator known to enhance ERK phosphorylation [39]. Remarkably, Ro 67-7476 treatment reversed the C5aR1 knockdown-induced decreases in the protein levels of p-ERK1/2 and METTL3 in U87 cells (Fig. 7D). Therefore, we hypothesized that p-ERK1/2 is the link between C5aR1 and METTL3. This hypothesis was confirmed by Co-IP experiments in U87 cells (Fig. 7E). In addition, U87 cells were treated with cycloheximide (CHX) to block protein synthesis, after which METTL3 degradation was assessed over time (0 h, 3 h, and 6 h) in cells with and without C5aR1 knockdown. METTL3 was rapidly degraded after C5aR1 knockdown, while Ro 67-7476 treatment significantly delayed its degradation (Figs. 7F and S7).

## Discussion

**Fig. 8:**
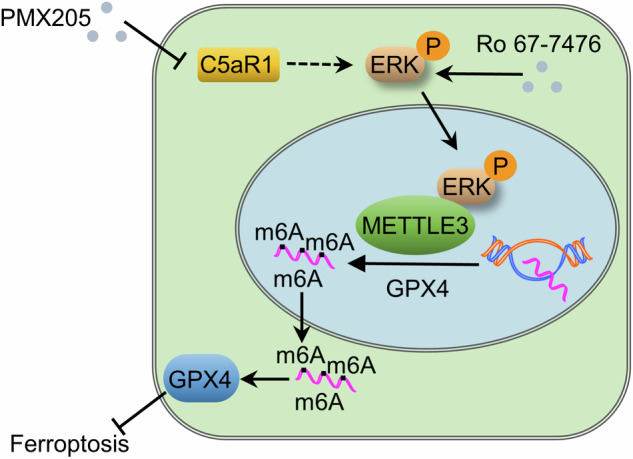
Proposed mechanism by which C5aR1 inhibits ferroptosis and promotes GBM progression. Intracellular C5aR1 activates the ERK1/2 signaling pathway, which transduces signals into the nucleus. These signals then increase METTL3 expression and stabilize the m6A level of GPX4, thereby upregulating GPX4 expression to prevent ferroptosis in GBM cells and ultimately promoting GBM progression. In addition, treatment with the p-ERK1/2 agonist Ro 67-7476, overexpression of METTL3, and overexpression of GPX4 strongly protect GBM cells from ferroptosis caused by C5aR1 knockdown.

Complement and complement receptors are becoming recognized as potential targets for molecular anticancer therapy [40]. They not only mediate the effects of tumor immunotherapy but also activate tumorigenic signaling pathways in tumor cells [30, 41]. In this study, we found that C5aR1, which is highly expressed in GBM and localized within GBM cells, can promote the development of GBM. This ability is largely due to its protective effects against lipid peroxidation and ferroptosis in GBM cells. Mechanistically, C5aR1 activates the ERK1/2 signaling pathway to transduce signals into the nucleus; then, these signals target METTL3 expression and stabilize the m6A level of GPX4, thereby upregulating GPX4 expression to prevent ferroptosis in GBM cells and ultimately promoting GBM progression (Fig. 8). We investigated the mechanism of action of C5aR1 in GBM and revealed a functional epigenetic signaling pathway that regulates ferroptosis. This pathway may provide a new target for gene therapy of GBM.

Ferroptosis is the most active programmed cell death process in glioma cells [42]. Glioma cells are highly sensitive to ferroptosis, and ferroptosis induction has been shown to enhance tumor cytotoxicity, thereby inhibiting GBM progression [43]. In addition, glioma cells possess a robust capacity for lipid synthesis, which continuously supplies the necessary material and energy for their malignant proliferation [44]. This ability is enhanced with increasing degree of glioma malignancy. Therefore, limiting lipid synthesis and inducing lipid peroxidation in GBM cells is a promising treatment strategy [45]. As previously shown, CDKN2A deletion reprograms lipid metabolism, leading to ferroptosis in GBM cells [5], and Rho family GTPase 1 promotes ferroptosis in GBM cells through p53 [46]. In this study, we focused on GBM cells and found that intracellular C5aR1 promotes GBM development by increasing GPX4 expression and thereby preventing ferroptosis in a GPX4-dependent manner. Our results are consistent with those of previous studies and indicate that ferroptosis induction is an effective therapeutic strategy for GBM.

One important finding of our research is that during the progression of GBM, the aberrant increase in C5aR1 expression is closely related to ferroptosis induction in GBM cells through targeting of METTL3 and subsequent METTL3-mediated regulation of GPX4 transcription. The malignant transformation of GBM is influenced by various epigenetic modifications, including DNA methylation and m6A modification [16, 17]. GPX4, a key molecule in ferroptosis, is subject to epigenetic modification [37]. Currently, synthetic small molecule compounds and natural plant extracts, such as RSL3, terpenoids, and phenols, have been developed to induce ferroptosis for cancer treatment by directly inhibiting GPX4, but these experimental drugs typically exhibit severe cytotoxicity and have unclear pharmacokinetic profiles [47–49]. Thus, their clinical translation is challenging. We found that C5aR1 knockdown significantly inhibited GPX4 at the transcriptional level and that overexpression of METTL3 significantly reversed the decrease in GPX4 expression and the induction of lipid peroxidation, and ferroptosis. Furthermore, si-METTL3 effectively inhibited GPX4 expression. Since the mechanisms by which epigenetic modifications affect the expression of specific genes are interconnected, our study not only reveals important epigenetic features of GPX4 inhibition and GBM cell ferroptosis but also suggests that the anti-GBM effects of GPX4 inhibition and ferroptosis can be achieved through intervention with METTL3 and potentially other molecules mediating epigenetic regulation.

One intriguing observation from our study is that C5aR1 is involved in regulating METTL3-dependent GPX4 transcription via the ERK1/2 pathway. Previous studies have shown that ERK1/2 pathway activity promotes glioma progression and that phosphorylated (active) ERK1/2 can continuously activate downstream targets and promote tumorigenesis [50]. For example, GBP5 drives GBM malignancy through the ERK1/2 pathway [51], and LY6K promotes the tumorigenicity of GBM cells through enhancement of ERK1/2 signaling [52]. We found that after C5aR1 knockdown, p-ERK1/2 directly bound to METTL3 in the nucleus and regulated its protein stability to regulate the m6A methylation of GPX4. This effect may be mediated through the activation of phosphorylation signals to regulate the phosphorylated site in METTL3 [53]. However, further investigations are needed to confirm this speculation. In addition, the p-ERK1/2 agonist Ro 67-7476 significantly reversed the decrease in METTL3 expression caused by C5aR1 knockdown. These data suggest that p-ERK1/2 are other important regulators of GBM progression. After C5aR1 knockdown, p-ERK1/2 targets METTL3 and regulates the m6A level of GPX4, thereby sensitizing GBM cells to ferroptosis.

Importantly, our study has several limitations. For example, the pharmacokinetic profile of PMX205 is unclear in an intracranial xenograft mouse model, and further investigation is required to elucidate the precise mechanism by which C5aR1 impacts GSH levels. In addition, the development of chemoresistance is a serious problem in the clinical treatment of GBM, and whether C5aR1 can reduce chemoresistance in GBM is another interesting but unanswered question.

In summary, we found that the transcription of GPX4 driven by C5aR1 is an epigenetic element of ferroptosis regulation in GBM and has important implications for the progression of GBM. C5aR1, a receptor for complement C5a, has been primarily studied for its immunoregulatory function. In this study, we found for the first time that C5aR1 in GBM cells can regulate ferroptosis through an epigenetic mechanism. Since epigenetic modifications and some regulated cell death programs are reported to be reversible and epigenetic drugs are emerging as effective clinical options, our findings provide strong evidence for therapeutic applications. Our study shows that a strategy targeting C5aR1 can be used to treat GBM by suppressing the epigenetic program of METTL3-dependent m6A modification of GPX4.

## Supplementary information

supplementary figure

original image

## Data Availability

All original data or reasonable requests reported in this paper are available from the corresponding author upon request.
