# Supplementary material for: Intracellular C5aR1 inhibits ferroptosis in glioblastoma through METTL3-dependent m6A methylation of GPX4
Source: Cell Death Dis. 2024 Oct 5;15(10):729. doi: 10.1038/s41419-024-06963-5 (PMC11455874; doi:10.1038/s41419-024-06963-5)
Supplement: Supplementary file 1 — supplementary figure [file 41419_2024_6963_MOESM1_ESM.pdf]

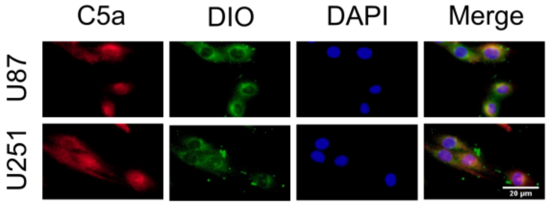

Figure S1. Immunostaining of U87 cells and U251 cells with antibodies against C5a (red) and cell membrane (green). Scale bar, 20 μm.

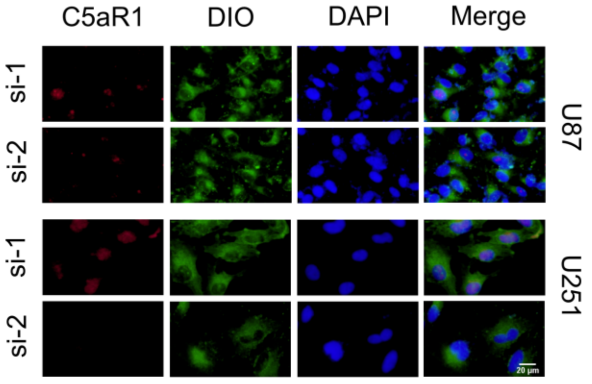

Figure S2. Immunostaining of U87 cells and U251 cells with antibodies against C5aR1 (red) and cell membrane (green). Knockdown C5aR1 decreased C5aR1 levelsin intracellular. Scale bar, 20 μm.

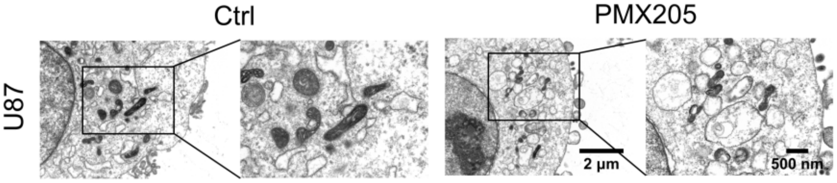

Figure S3. The mitochondria of U87 cells treated with PMX205 were observed by transmission electron microscopy. Scale bar, 2 μm and 500 nm.

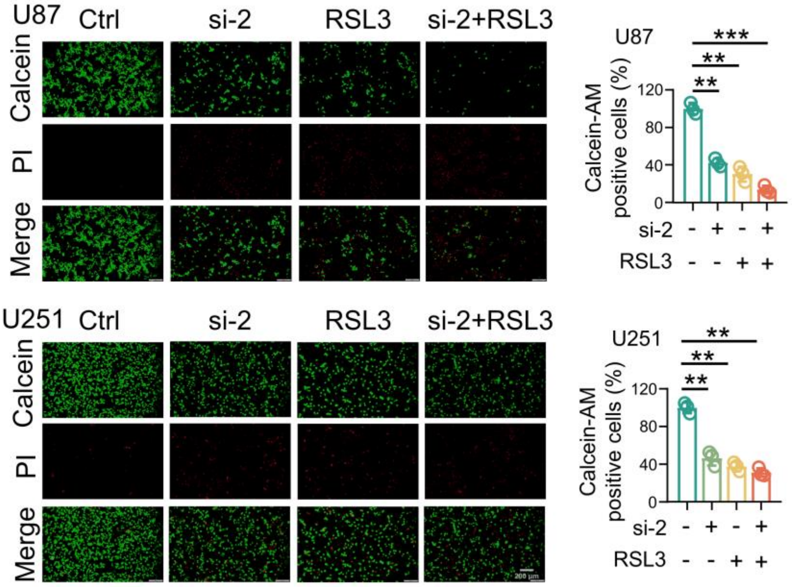

Figure S4. Calcein AM/PI staining of U87/U251 cells after C5aR1 knockdown and/or RSL3 treatment was detected via fluorescence microscopy. Scale bar, 200 μm.

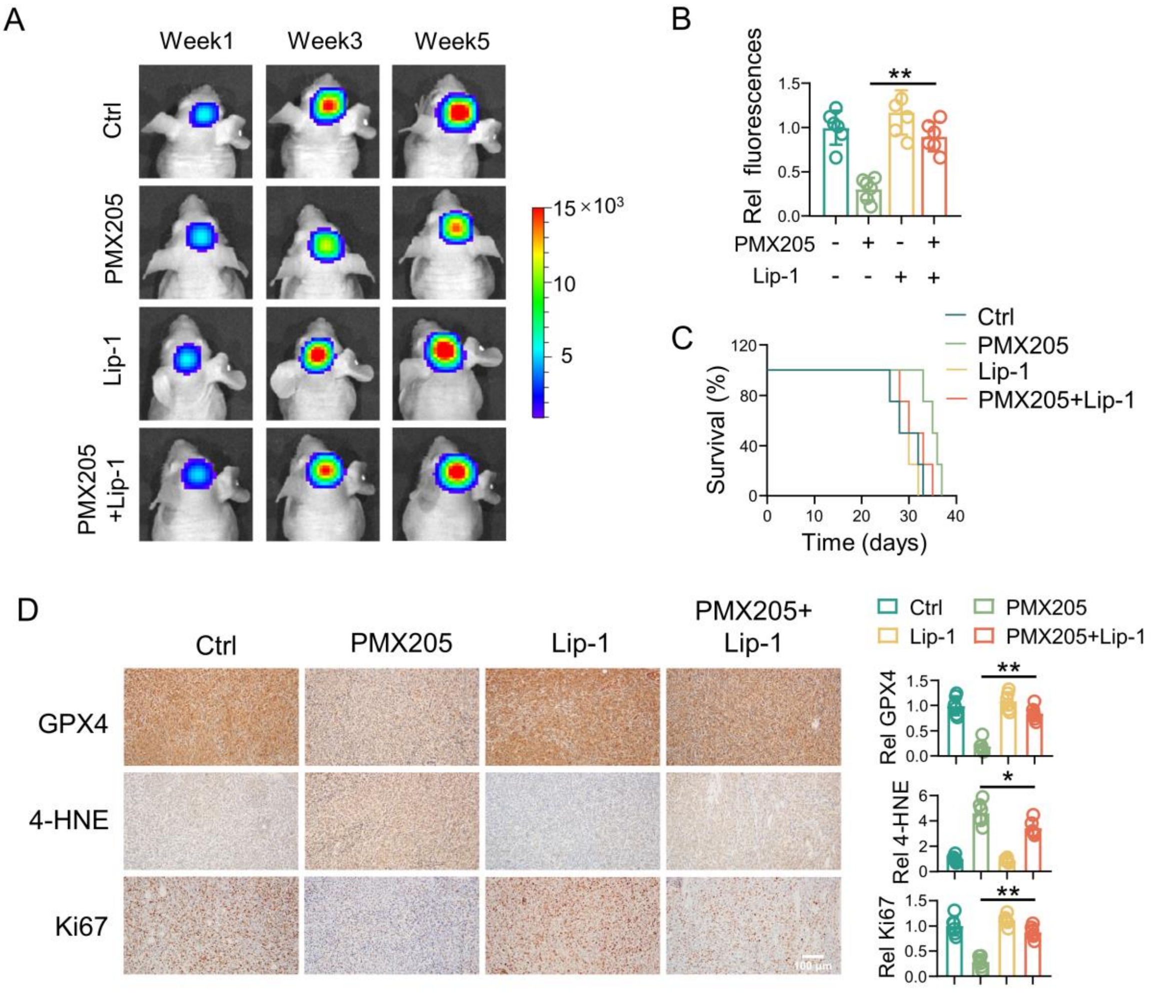

Figure S5. A Representative bioluminescence images showing luciferase signals in tumors in mice in the different treatment groups at 1, 3 and 5 weeks acquired by IVIS imaging. **B** Quantitative analysis of the data in A. **C** Mouse survival is shown on Kaplan–Meier curves (n=6). PMX205 group was compared with PMX205+Lip-1 group, *P*=0.0446. **D** IHC staining of GPX4, 4-HNE and Ki67 in tumor tissue, and the related quantitative analysis. Scale bars, 100 μm.

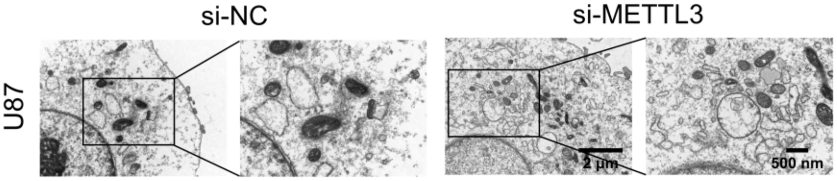

Figure S6. The mitochondria of U87 cells treated with si-METTL3 were observed by transmission electron microscopy. Scale bar, 2 μm and 500 nm.

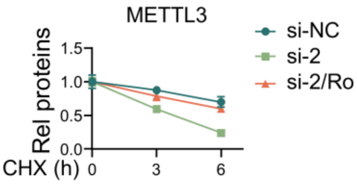

Figure S7. The results of Figure 7F were quantitatively analyzed.
