# Supplementary material for: Intracellular C5aR1 inhibits ferroptosis in glioblastoma through METTL3-dependent m6A methylation of GPX4
Source: Cell Death Dis. 2024 Oct 5;15(10):729. doi: 10.1038/s41419-024-06963-5 (PMC11455874; doi:10.1038/s41419-024-06963-5)

# WB original image

## Fig 1 E

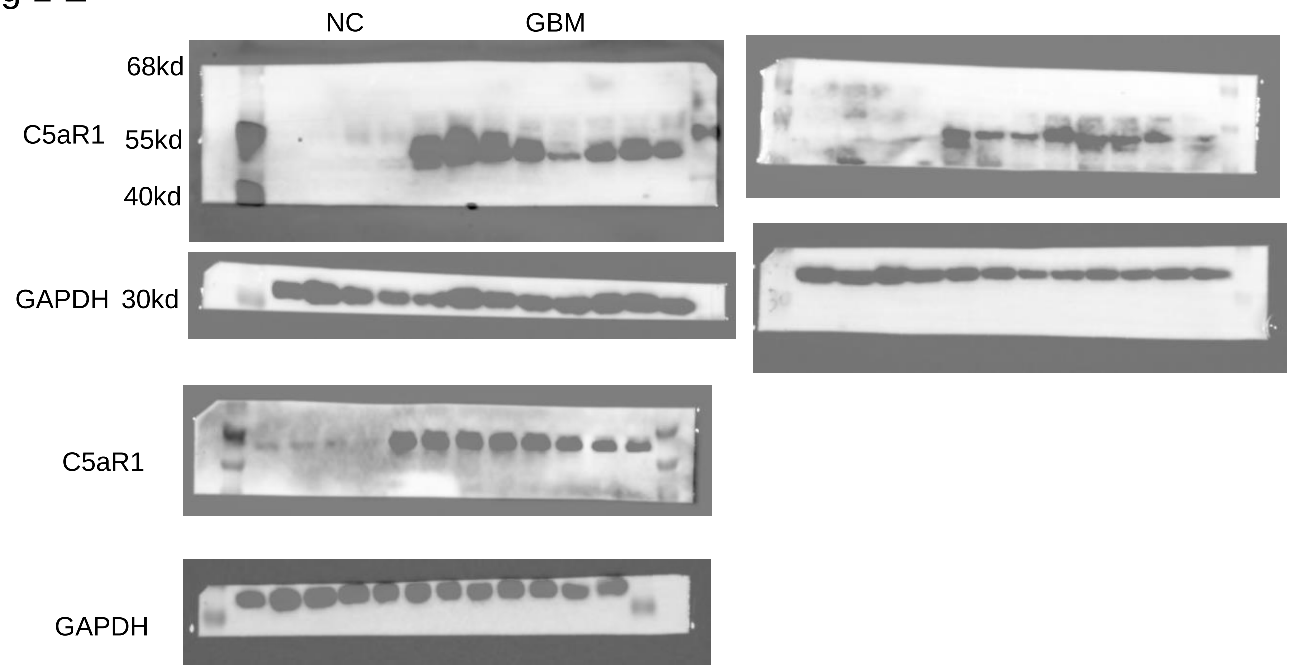


## Fig 1 G

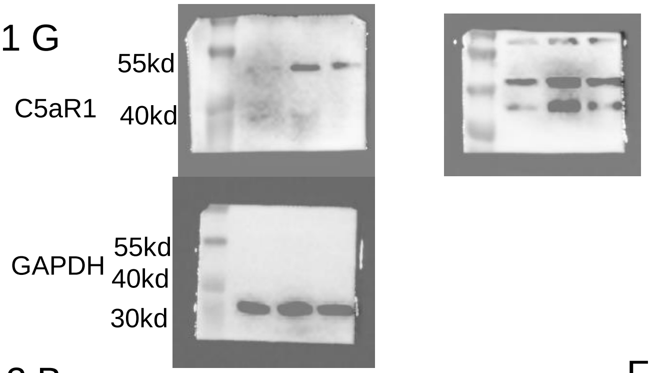


## Fig 2 B

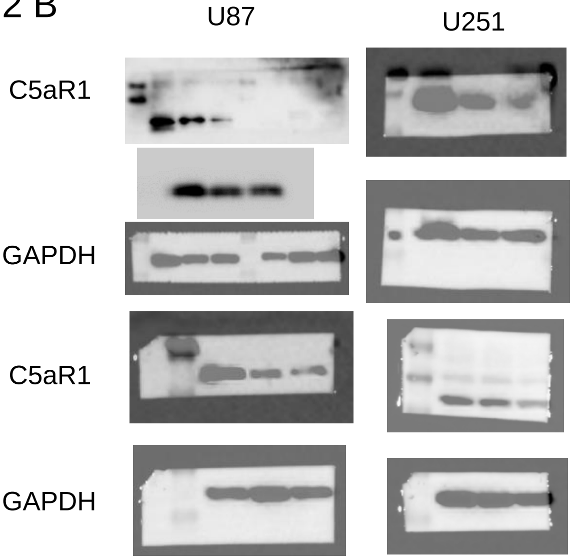


## Fig 3 B

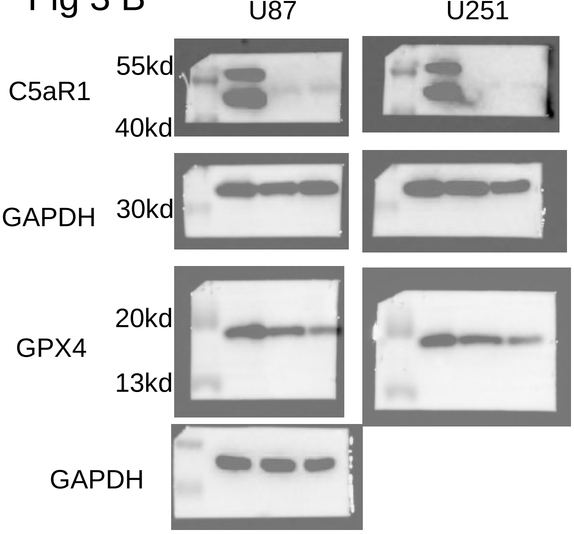

## Fig 3 C

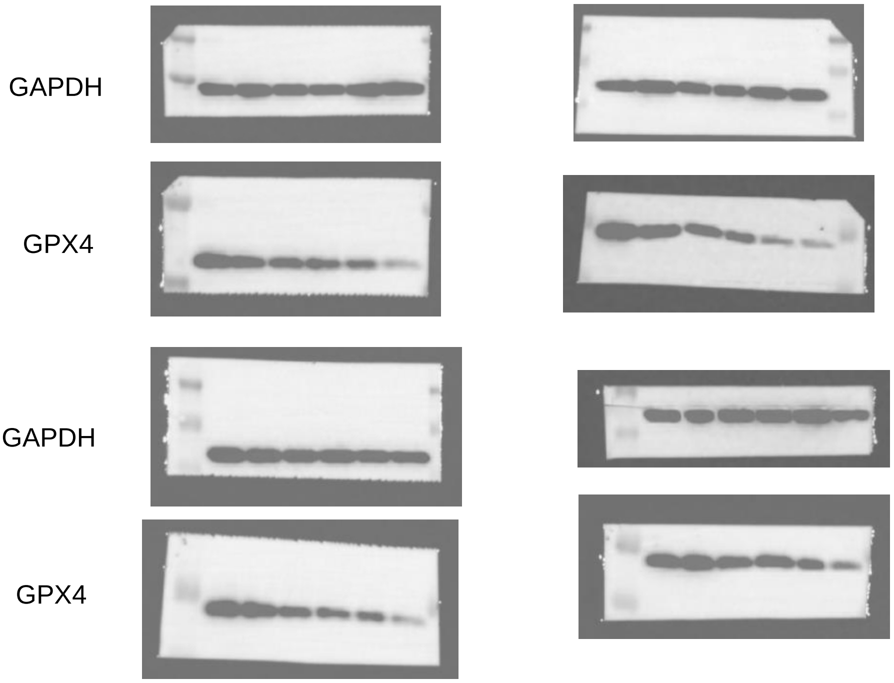


## Fig 3 D

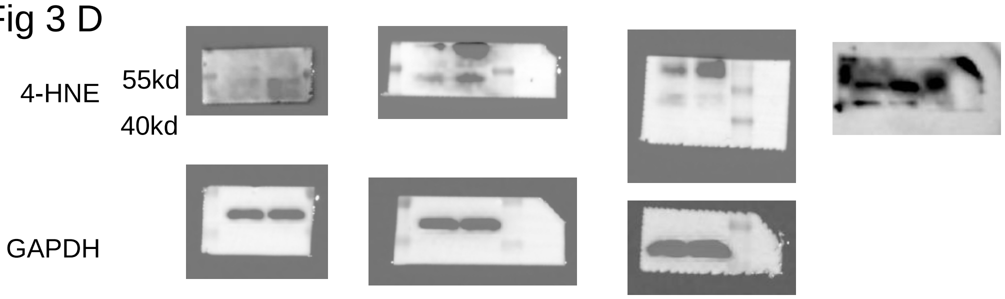


## Fig 4 E

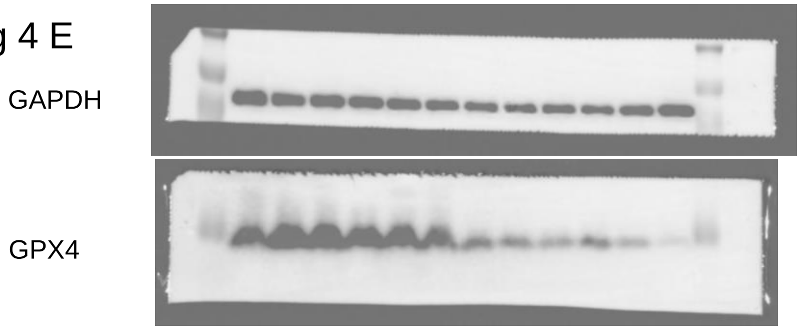


## Fig 5 A

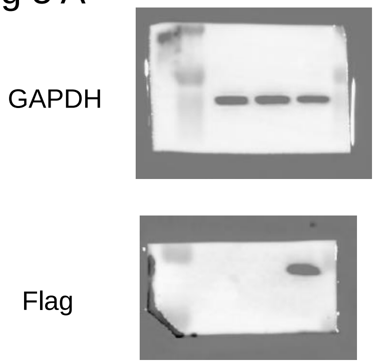


## Fig 5 C

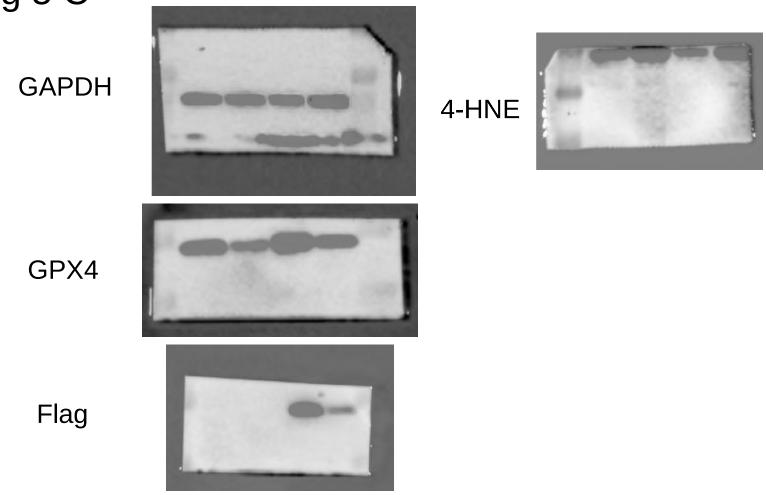

Fig 6 D

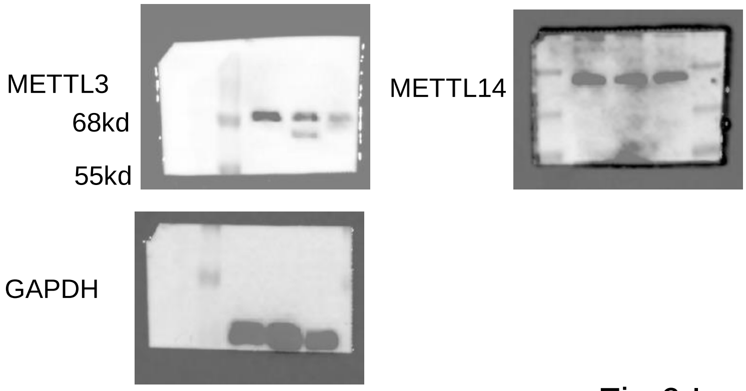


Fig 6 F

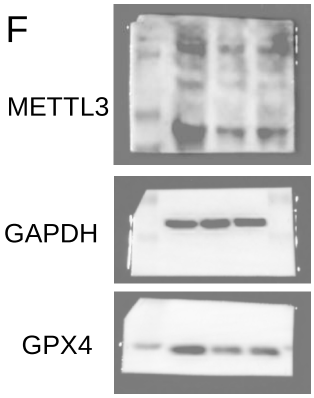


Fig 6 I

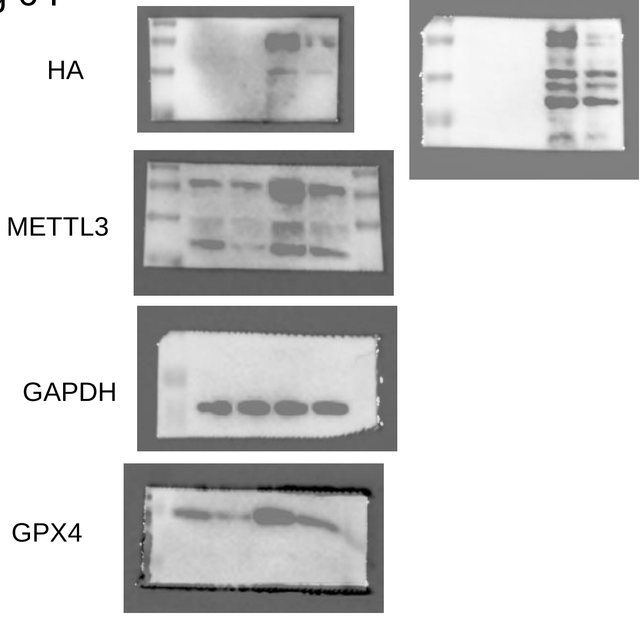


Fig 6 G

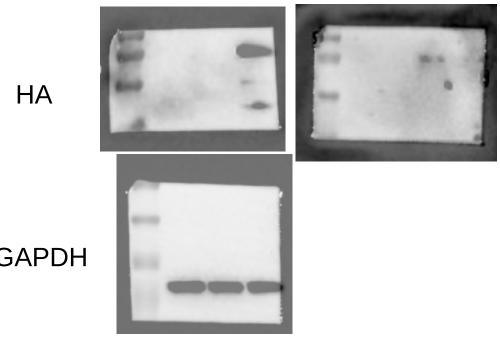


Fig 7 B

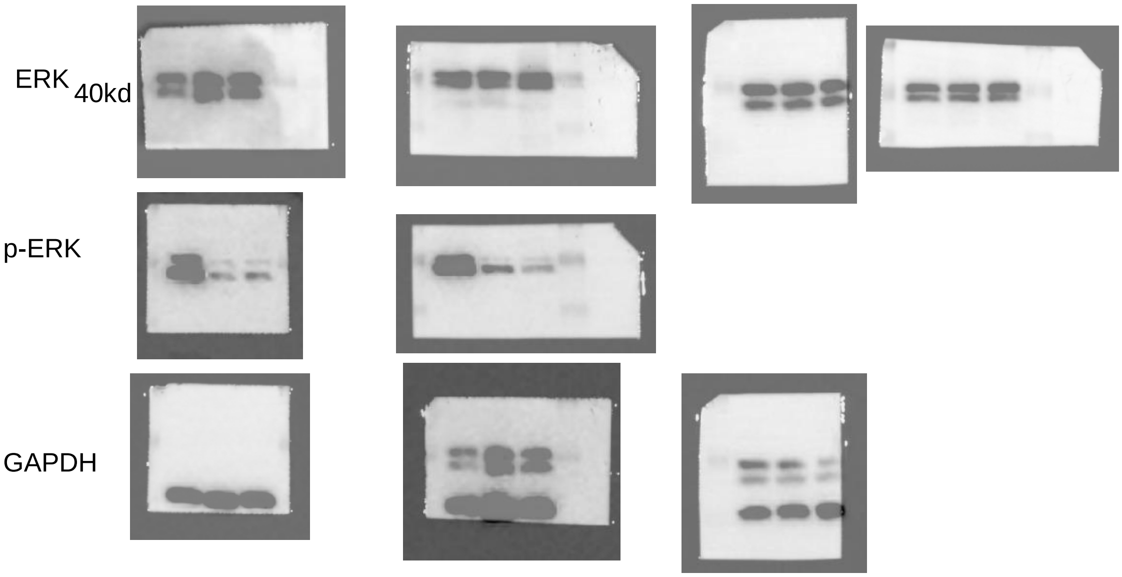

Fig 7 D

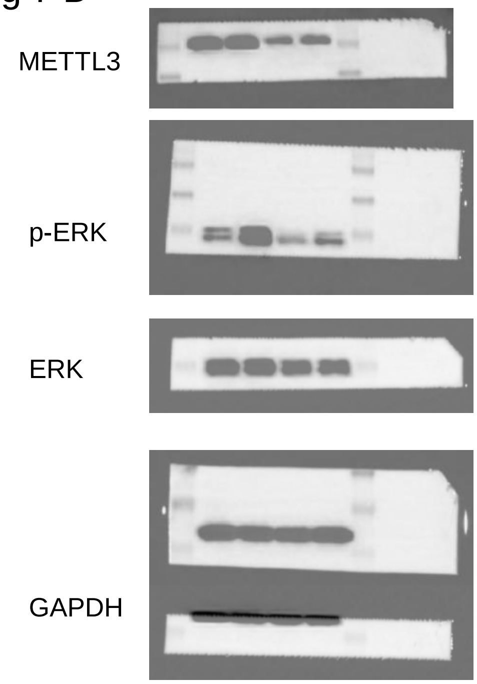


Fig 7 E

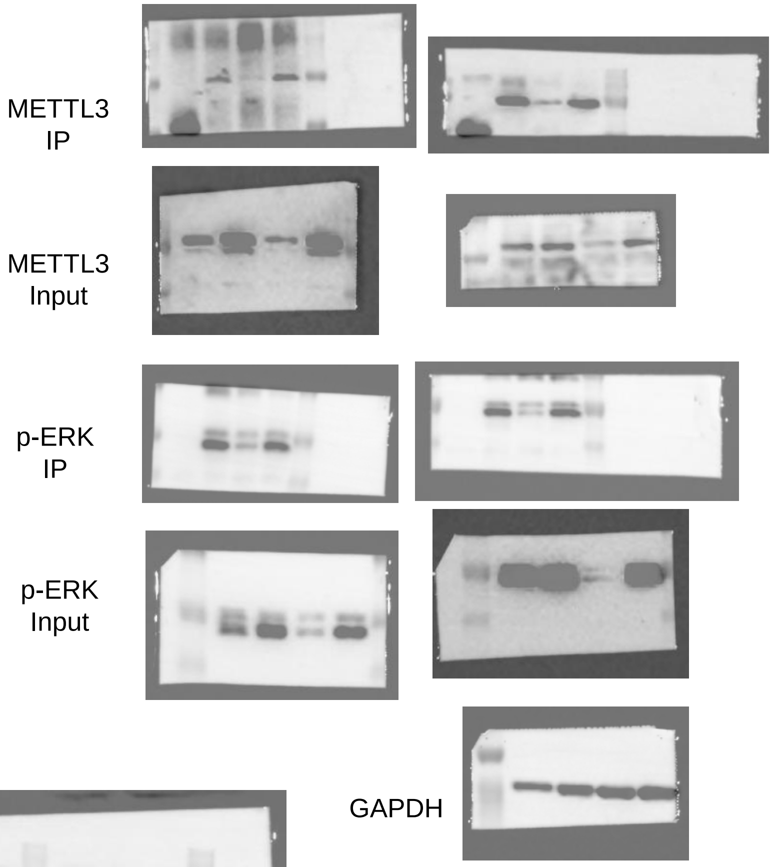


Fig 7 F

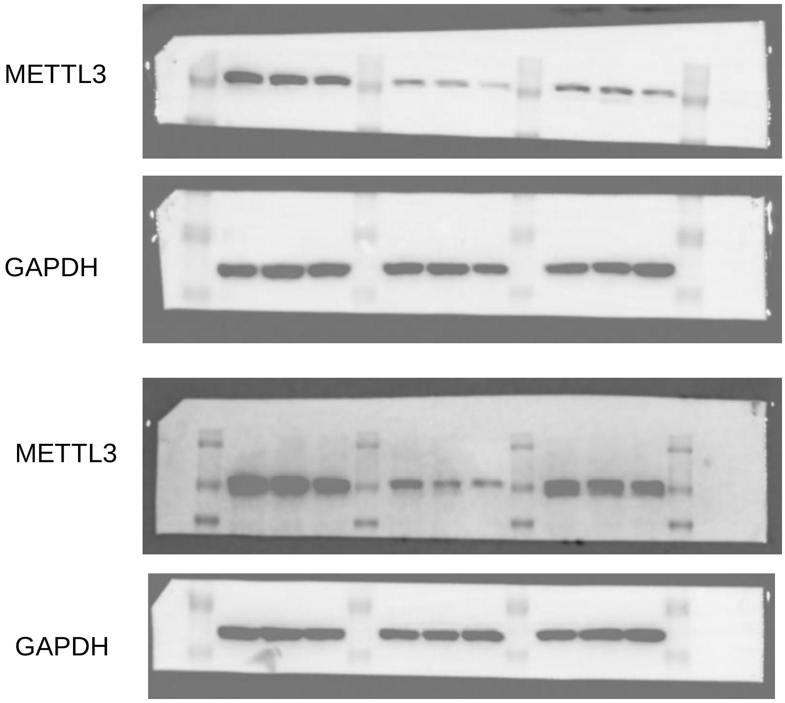

Supplement: Supplementary file 2 — original image [file 41419_2024_6963_MOESM2_ESM.pdf]
